# Application of digital health interventions in the management of economic toxicity in cancer patients: a scoping review

**DOI:** 10.3389/fpubh.2026.1831148

**Published:** 2026-04-21

**Authors:** Ziyi Chen, Bixuan Xu, Jinpeng Wen, Shijie Zhao, Yuling Zheng, Hejia Wan, Juntao Wang

**Affiliations:** 1 Department of Oncology, The Third Affiliated Hospital of Henan, University of Chinese Medicine, Zhengzhou, Henan, China; 2 Cancer Diagnosis and Treatment Center, The First Affiliated Hospital of Henan University of Chinese Medicine, Zhengzhou, Henan, China; 3 School of Nursing (Nursing School of Smart Healthcare Industry), Henan University of Chinese Medicine, Zhengzhou, Henan, China; 4 School of Journalism and Communication, South China University of Technology, Guangzhou, Guangdong, China

**Keywords:** cancer care, digital health, economic toxicity, financial navigation, health equity, scoping review, supportive care, telehealth

## Abstract

**Objective:**

This study systematically reviews the current application of digital health interventions in cancer patients, clarifies their implementation characteristics and intervention effects in managing economic toxicity, and provides a reference for intervention programs to reduce the economic toxicity of patients.

**Methods:**

Relevant studies were systematically retrieved from PubMed, Web of Science, Cochrane Library, Embase, CINAHL, CNKI, CBM, WanFang Database, and VIP Database from their inception to February 6, 2026. Data from the included literature were extracted and analyzed.

**Results:**

A total of 22 studies were included. Digital health technologies are applicable to multiple stages of cancer patient diagnosis, treatment, and rehabilitation. Among these, remote financial navigation or consultation is the most commonly used intervention method. Existing research has shown positive results in improving cost communication skills, facilitating financial assistance, and enhancing intervention feasibility, but evidence regarding improvements in economic toxicity scales and the alleviation of long-term financial difficulties exhibits some heterogeneity.

**Conclusion:**

Digital health technologies, with their high accessibility, scalability, and ease of remote access and follow-up, offer unique advantages in managing the economic toxicity of cancer patients. Current research in this field has gradually shifted from single-stage interventions to comprehensive intervention pathways. Future research should focus on the dynamic evolution of economic toxicity at different stages of diagnosis and treatment, promote the standardization of intervention procedures and outcome evaluations, and develop personalized digital intervention strategies to mitigate the economic toxicity of cancer patients.

**Systematic review registration:**

https://doi.org/10.17605/OSF.IO/4DYN3.

## 1 Introduction

According to global cancer statistics released by the International Agency for Research on Cancer (IARC), there were approximately 19,976 million new cancer cases and 9,744 million cancer deaths worldwide in 2022. With increasing population aging and growth, the number of new cancer cases globally is projected to exceed 35 million by 2050, an increase of approximately 77% compared to 2022 (1–3). Cancer, as a major chronic and life-threatening disease, imposes a significant burden on individuals and society (4, 5). With the continuous improvement of comprehensive treatment levels, the survival time of cancer patients continues to extend, gradually showing a trend towards chronic disease status and long-term management (6). Against this backdrop, cancer patients’ concerns have further expanded to include symptom management, quality of life, and the burden of long-term care (7). Treatment-related economic burdens have long been insufficiently incorporated into routine cancer management systems, but they do have a certain degree of impact on patients’ treatment experience, care decisions, and prognostic outcomes (8, 9).

The concept of economic toxicity was first proposed by Zafar and Abernethy in 2013 and has been applied in the field of oncology (10). It refers to the negative psychological, material, and behavioral effects of the high costs of disease treatment on patients and their families, mainly including objective economic burden and subjective economic distress. The National Cancer Institute points out that (9) economic toxicity not only creates debt and bankruptcy risks, but may also affect patients’ ability to access medical services, causing them to postpone medical treatment, reduce medication, or abandon some treatments due to cost reasons. Existing research further indicates that (11) economic toxicity is closely related to decreased quality of life, increased psychosocial burden, reduced treatment adherence, and adverse clinical outcomes, and is an important non-biological factor affecting the quality of cancer management throughout the entire process. Therefore, it is crucial to identify high-risk groups for the economic toxicity of cancer patients, mitigate the economic risks of relevant vulnerable groups, and implement effective and feasible interventions.

Digital health interventions (DHIs) refer to interventions based on digital technologies, including those based on web platforms, mobile applications, social media, or wearable devices (12). Multiple studies have shown that (13–15) DHIs can effectively improve cancer-related symptoms and quality of life, and reduce anxiety, depression, and suffering. Regarding the economic toxicity of cancer patients, digital health technologies can achieve early identification of financial risks through electronic screening, patient-reported outcomes, and intelligent stratification. By providing financial guidance, insurance education, resource matching, and ongoing follow-up via telephone, video, online platforms, or mobile tools, these technologies can alleviate the economic burden and treatment costs for cancer patients to some extent (16, 17). While existing research has provided a general overview of interventions for the economic toxicity of cancer (18), the integration of intervention models, implementation methods, and application pathways from a “digital health” perspective remains relatively insufficient. A comprehensive review is necessary to synthesize existing evidence and practical progress (19). Scope reviews, as an important form of review used to systematically review the current state of research, integrate different types of evidence, and identify knowledge gaps, have been widely used in many research fields such as symptom management, critical care nursing, and advanced practice nursing in recent years (4, 5, 20, 21). Therefore, this study adopts a comprehensive review approach to systematically review the current application status of digital health interventions in the management of economic toxicity in cancer patients, focusing on summarizing their basic components and intervention forms, and summarizing their application effects in improving the economic toxicity of cancer patients, providing a reference for subsequent intervention program development, clinical practice optimization, and related research design.

## 2 Materials and methods

### 2.1 Defining the research question

This scoping review was conducted following the “PCC” methodological framework published by the Joanna Briggs Institute (JBI) (22) in Australia. The object of this research review is cancer patients; the concept is the application of digital health interventions in the management of economic toxicity in cancer patients; and the context is economic toxicity management services for cancer patients. Therefore, the main research questions of this study are: What are the basic components and intervention forms of the application of digital health technology in the management of economic toxicity in cancer patients? How effective are existing research reports on the application of digital health interventions in improving the economic toxicity of cancer patients?

### 2.2 Search strategy

The system searched PubMed, Web of Science, Cochrane Library, Embase, CINAHL, SinoMed, CNKI, Wanfang Database, and VIP Database, with a timeframe of database construction to February 2026. The Chinese search focuses on cancer, economic toxicity, and digital health intervention. Keyword categories include

“tumor/cancer/malignant tumor/tumor patients”

“economic toxicity/financial toxicity/Economic burden/financial difficulties/economic pressure”

“digital health/electronic health/mobile health/telemedicine/internet healthcare”. Free keywords include “financial navigation/insurance navigation/cost communication/cost management/electronic screening/remote follow-up/online platform/mobile application/APP/digital intervention”; the English search terms are“cancer/neoplasm/malignancy/tumor”

“financial toxicity/financial hardship/financial burden/financial distress” “digital health/eHealth/mHealth/telemedicine/telehealth”

“financial navigation/insurance navigation/cost management” “electronic screening/electronic patient-reported outcomes”

“telephone follow-up/web-based intervention/mobile application/app/digital intervention”.

Searches were conducted using a combination of subject and free word and manual searches while snowballing through the included literature. The search timeframe was from database construction to February 6, 2026.

### 2.3 Literature inclusion and exclusion criteria

Inclusion criteria:

(1) The study subjects are cancer patients, without restrictions on age, gender and region;
(2) The intervention method is digital health technology, including but not limited to the use of mobile applications, online platforms, telephone or video follow-up, telemedicine or digital resource links and other related intervention measures;
(3) The report must include economic toxicity related outcome indicators, whether they are primary or secondary outcomes, they shall be included;
(4) The literature type is original research, including randomized controlled trials, experimental-like studies, mixed-method studies, case–control studies, cohort studies, longitudinal studies and case reports, etc.;
(5) The language is Chinese or English.

Exclusion criteria:

(1) The intervention method is a non-digital health intervention;
(2) Non-original research such as reviews, systematic reviews, conference abstracts, research protocols, editorials/comments;
(3) Duplicate publication;
(4) The full text is unavailable.

### 2.4 Literature screening and data extraction

The retrieved literature was imported into Zotero software for deduplication. Two researchers independently screened eligible studies based on inclusion and exclusion criteria, and then conducted a second screening based on the full text. Disagreements were resolved through discussion or by consulting a third researcher. A data extraction table was established for studies on the application strategies and management effects of digital health interventions in the economic toxicity management of cancer patients. Two researchers independently extracted basic information and data from the included literature. Disagreements or questions during the extraction process were resolved through consultation with a third researcher. Extracted information included first author, country of publication, publication date, study design, study population, sample size, intervention measures, intervention effects, and outcome indicators.

## 3 Results

This section may be divided by subheadings. It should provide a concise and precise description of the experimental results, their interpretation, as well as the experimental conclusions that can be drawn.

### 3.1 Results of literature search and screening

**Figure 1:**
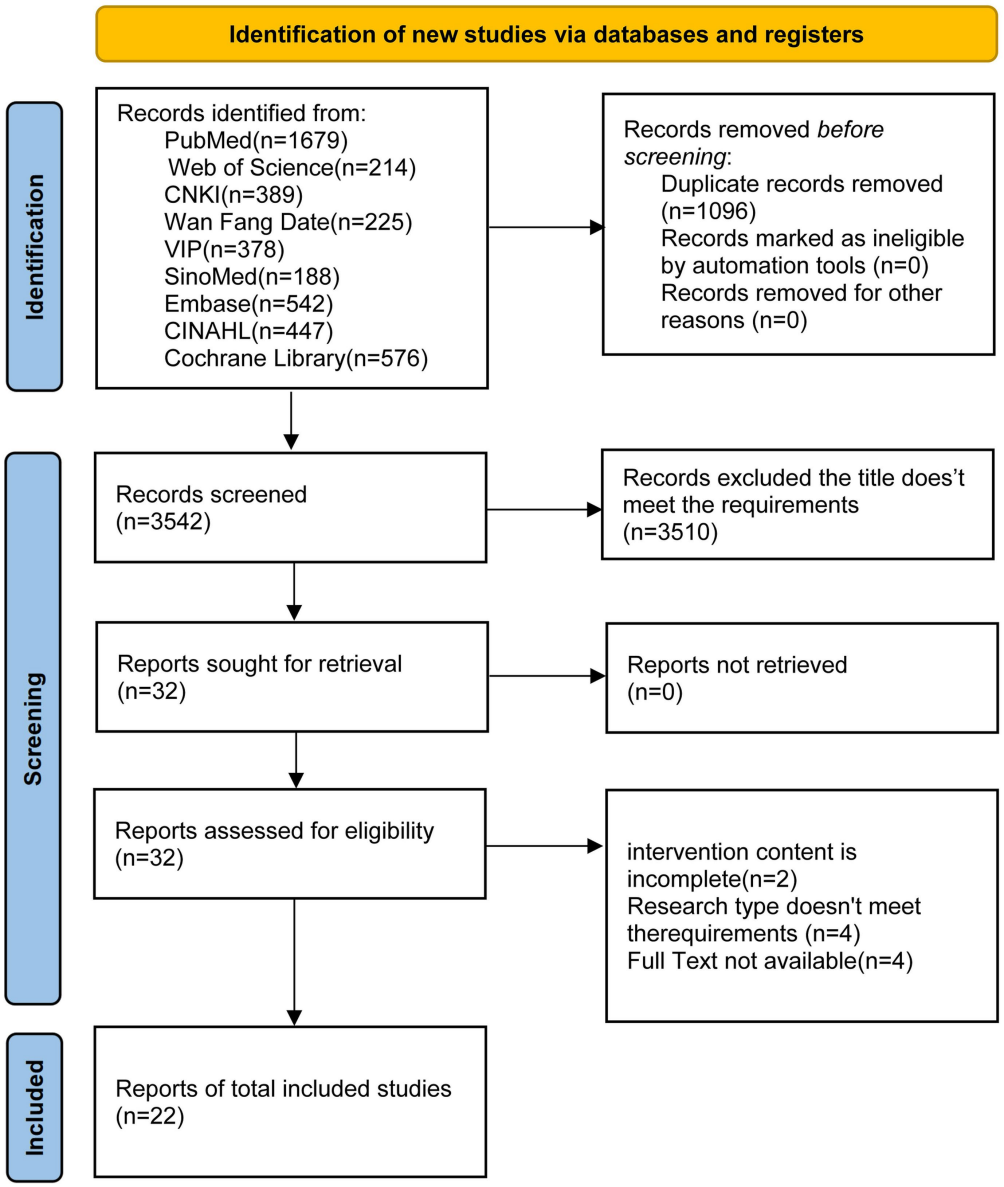
Literature retrieval and screening process.

The preliminary search yielded 4,638 documents, with 3,542 documents remaining after de-weighting, and 22 documents were finally included after reading the titles, abstracts, and full texts (23–44), of which 1 were in Chinese (23), and 21 were in English (24–44). The flowchart of literature screening is shown in Figure 1. The basic characteristics of the included literature are shown in Table 1.

**Table 1:** The basic characteristics of the included literature (*n* = 22).

#	Author	Country	Date	Study design	Cancer types	Sample size (IG/CG; total)	Interventions	Intervention effect	Outcome indicators
1	Song et al. (23)	China	2025	Quasi-experimental study	Prostate cancer	30/30;60	Nurse-led navigation plus resource support; comparator: routine care	The intervention group showed a greater reduction in financial toxicity at discharge and at 3 months after discharge (*p* < 0.05)	COST-PROM; adherence/compliance behavior questionnaire
2	Alacevich et al. (24)	United States	2024	RCT	Unlimited	40/41/40;121	Telehealth financial counseling (individual or group, via Zoom, ≥2 sessions); comparator: usual care plus booklet	COST scores generally improved from baseline, but no clear statistically significant between-group difference was observed	COST; TUQ; feasibility metrics
3	Bell-Brown et al. (25)	United States	2024	RCT	Stomach cancer	10/9;19	Proactive financial navigation (CENTS plus PAF; monthly for 3 months); comparator: usual care	The intervention reduced household financial hardship within 6 months, but subjective financial stress was similar between groups	Household financial hardship; FACT-G; COST-FACIT; qualitative assessment of usual care/intervention; City of Hope QOL; caregiver burden
4	Wheeler et al. (26)	United States	2022	Case study	Unlimited	30	LIFT financial navigation model (financial toxicity screening, one-to-one navigation, and issue tracking; biweekly for up to 6 months); no comparator	The study clarified the core intervention and implementation functions of the LIFT financial navigation model, supporting adaptation and scale-up in oncology settings	Core intervention functions; implementation functions; workflow characteristics; barriers and facilitators; adaptation and scale-up considerations
5	Blinder et al. (27)	United States	2023	RCT	Metastatic cancer	593/598; 1,191	Digital PRO monitoring plus monthly financial toxicity screening and nurse alerts; comparator: usual care	Embedding financial toxicity screening within digital PRO monitoring reduced the incidence or worsening of financial hardship (*p* = 0.004)	EORTC QLQ-C30 financial difficulties item; FACIT-COST single-item screener; weekly digital PRO surveys; nurse alert responses/actions; patient interviews; nurse interviews
6	Budhu et al. (28)	United States	2025	Quasi-experimental study	Breast, gynecological, gastroenterologi-cal, and thoracic oncology	50,949	Electronic screening (FACIT-COST plus HRSR) with patient financial services outreach/referrals; no comparator	Electronic screening identified patients needing help, but substantial attrition occurred along the referral pathway from positive screening to receipt of assistance	FACIT-COST; HRSR checklist; new borrowing/loans for treatment; positive screening rate; documented outreach; successful contact; documented intervention receipt; intervention types; barriers
7	Edward et al. (29)	United States	2024	Quasi-experimental study	Unlimited	15	Financial–legal navigation program, including social work screening, financial navigation, and legal support (5–20 contacts); no comparator	The intervention improved financial toxicity in adult patients and caregivers, showed high retention, and generated substantial economic benefits	FACIT-COST; MEPS-ECSS items; debt/borrowing; bankruptcy indicators; coping behaviors; total financial toxicity score; PROMIS Global Health; PROMIS Anxiety; PROMIS Depression; economic benefits; feasibility; appropriateness;et al
8	Edward et al. (30)	United States	2023	Quasi-experimental study	Hematologic malignancies	54	Embedded financial navigation program (CC Links), including COST/DT screening and benefits/financial assistance support; no comparator	After the intervention, the psychological response domain of financial toxicity significantly improved, and the intervention showed high acceptability	COST; MEPS-ECSS material conditions items; MEPS-ECSS coping behaviors; NCCN Distress Thermometer; PROMIS Global Health; PROMIS Anxiety; financial benefits secured; acceptability
9	Elshafie et al. (31)	United States	2025	Quasi-experimental study	Breast cancer	874/267;1,141	Telehealth use vs. nonuse (claims-based cohort)	Associated with better endocrine therapy adherence (*p* < 0.001), but higher out-of-pocket medical expenses	Endocrine therapy adherence; Metastasis incidence; Out-of-pocket medical costs; Out-of-pocket prescription costs; Telehealth visits
10	Hamel et al. (32)	United States	2022	Quasi-experimental study	Breast/Lung/Prostate/Colorectal Cancer	32	DISCO App tailored cost QPL + physician tip sheet (single pre-visit use)	Improved self-efficacy in managing treatment costs and communicating with physicians (*p* < 0.001); cost-related distress decreased, but not significantly	Self-efficacy; Cost-related distress; Presence of cost discussion; Cost discussion initiator; Cost discussion topics; Feasibility; Acceptability ratings
11	Henrikson et al. (33)	United States	2025	RCT	Unlimited	123/127/121; 371	CAFÉ financial navigation (outreach plus 6-month navigation; brief = 1 outreach, extended = 3 outreaches); comparator: resource list only	No significant differences were found in financial distress or cancer-related quality of life, although participants generally considered financial navigation valuable	InCharge Financial Distress/Financial Well-Being Scale; FACT-G7; PROMIS Global Health; PACE; COST-FACIT; use of support services; intervention fidelity; exit interview; etc.
12	Jang et al. (34)	Taiwan, China	2024	Quasi-experimental study	End-stage cancer	293/293;586	Tele-assisted home palliative care (24/7 tele-support); comparator: no THPC	THPC was associated with fewer ED visits, hospitalizations, and ICU admissions before death, and with lower NHI expenditures	Outpatient visits; ED visits; Hospitalizations; ICU admissions; NHI reimbursements; Out-of-pocket medical expenditures; Estimated transportation costs; etc.
13	Khurana et al. (35)	United States	2025	Quasi-experimental study	AYA Cancer	23	AYA-NAV (financial toxicity and HRSN screening plus PAF/findhelp and monthly check-ins for 6 months); no comparator	COST scores improved significantly (*p* = 0.024), and most participants considered the intervention acceptable and helpful	COST; HRSN screening; feasibility; acceptability; appropriateness; fidelity; CD-RISC-10; PROMIS Global Health
14	Kirchhoff et al. (36)	United States	2023	RCT	AYA Cancer	45/41;86	HIAYA CHAT navigator-led insurance education (4 Zoom sessions); comparator: usual navigation	The intervention improved health insurance literacy and ACA knowledge at 5 months, but no significant between-group difference in COST scores was observed	Health Insurance Literacy; Insurance terminology knowledge; ACA protections knowledge; Federal insurance/employment protections familiarity; COST; Perceived Stress Scale; etc.
15	Lambert et al. (37)	United States	2019	Quasi-experimental study	Unlimited	Analyzed = 4,616; high-priority = 244; received assistance = 181	TailorMed technology-enabled financial navigation (EHR-linked matching plus navigator outreach); no comparator	The platform identified high-priority patients and facilitated financial assistance, resulting in substantial approved savings.	High-priority patients identified; Assistance uptake rate; Approved savings; Community benefit; Revenue increase; Assistance categories; etc.
16	Park et al. (38)	United States	2025	RCT	Colorectal cancer	17/19;36	HINT-C insurance navigation (5 Zoom sessions) plus manual; comparator: manual only	HINT-C was feasible and acceptable, and improved health insurance literacy and ACA knowledge, while improvements in financial burden were limited	Health Insurance Literacy; ACA knowledge; Familiarity with health care legislation; Psychological financial burden; Behavioral financial burden; Program satisfaction; etc.
17	Patel et al. (39)	United States	2026	Cross-Sectional Study	Unlimited	4,937	Employer-sponsored virtual navigation service (app, web, phone, and chat; utilization study); no comparator	Most chat users raised financial issues, and those reporting financial stress were more likely to use additional support services	Navigation service utilization; Chat-derived financial concern level; Types of financial inquiry; Use of financial support services
18	Sadigh et al. (40)	United States	2022	Quasi-experimental study	Unlimited	20	CostCOM individualized out-of-pocket communication plus remote financial navigation/counseling (1 h within 2 weeks plus follow-up); no comparator	Patients’ financial worries decreased significantly (*p* < 0.001)	COST; Financial self-efficacy scale; HILM; Cost-related care nonadherence; Material financial hardship; Financial assistance application completion; Copay assistance enrollment; Cost savings; etc.
19	Shankaran et al. (41)	United States	2018	Quasi-experimental study	Non-metastatic cancer	34	Financial navigation program (education plus CENTS and PAF; monthly for up to 6 months); no comparator	The program provided practical assistance to most patients, but overall self-reported financial burden changed little	Program adherence/participation; Self-reported financial burden; Anxiety about costs; Financial hardship; etc.
20	Sadigh et al. (42)	United States	2019	Quasi-experimental study	Unlimited	12	Brain cancer financial navigation with referral to PAF case management (at least monthly for at least 6 months); no comparator	Most financial issues were resolved and debt relief was obtained, although COST scores did not improve significantly in the small subgroup with 3-month follow-up data	COST; Financial coping; Financial hardship; Care nonadherence due to cost; Program participation; Number of PAF contacts; Issues discussed; Issues resolved; Debt relief
21	Tarnasky et al. (43)	United States	2021	RCT	Unlimited	100/100;200	Bridge app assistance matching; comparator: generic financial resources website	Post-hoc analyses suggested that the intervention group was more likely to apply for and receive financial assistance	Out-of-pocket costs; FACT-COST; Awareness of financial resources; Survey completion/attrition; Applied for financial assistance; Received financial assistance
22	Yuan et al. (44)	China	2025	RCT	Breast cancer	13/13;26	China breast cancer financial navigation program (assessment, education, referral, monthly calls, and WeChat support); comparator: usual care	The intervention improved cost-related health literacy, but no significant between-group difference in COST-FACIT scores was found at 1 month	Cost-related health literacy; COST-FACIT; Material hardship; Employment change; Cost-related nonadherence; etc.

IG = intervention group; CG = control group; RCT = randomized controlled trial; COST = Comprehensive Score for Financial Toxicity; PROM = patient-reported outcome measure; TUQ = Telehealth Usability Questionnaire; NCCN=National Comprehensive Cancer Network; DT = Distress Thermometer; FACT-G = Functional Assessment of Cancer Therapy-General; FACIT = Functional Assessment of Chronic Illness Therapy; ACA = Affordable Care Act; ED = emergency department; ICU = intensive care unit; HILM = Health Insurance Literacy Measure; PROMIS=Patient-Reported Outcomes Measurement Information System; OOP = out-of-pocket.

### 3.2 Basic characteristics of included literature

Twenty-two papers published between 2018 and 2026 were included. Existing research findings are mainly concentrated in the United States, with a limited number of studies in Asia. Among them, 19 were from the United States (24–33, 35–43), 2 from China (23, 44), 1 from Taiwan, China (34). The types of studies included in the literature were 8 randomized controlled trials (24, 25, 27, 33, 36, 38, 43, 44), 12 quasi-experimental studies (23, 28–32, 34, 35, 37, 40–42), 1 cross-sectional study (39) and 1 case study (26).

### 3.3 Research subjects of included literature

All study subjects were cancer patients. Among them, there were 7 studies targeting a single cancer type (23, 25, 29–31, 38, 42), including 2 studies on breast cancer patients (29, 31), 1 study on colorectal cancer patients (38), 1 study on prostate cancer patients (23), 1 study on gastric/gastroesophageal junction adenocarcinoma patients (25), 1 study on brain tumor patients (42), and 1 study on hematologic malignancies (30); 13 studies on patients with undetermined cancer types (24, 26–28, 33–37, 39–41, 43); and 2 studies that included multiple patients with clearly defined cancer types (32, 44).

### 3.4 Intervention characteristics of digital health technologies in the management of economic toxicity in cancer patients

#### 3.4.1 Intervention methods

The intervention methods used in the study were diverse, including remote financial navigation or consultation, digital screening and automated referral, insurance navigation and health literacy enhancement, cost communication facilitation, mobile applications or digital platforms, and telemedicine support.

(1) Remote financial navigation or remote financial consultation: among the 22 included studies, Eleven studies (23–25, 29, 30, 33, 35, 40–42, 44) used remote financial navigation or consultation as the primary intervention. Through telephone, video conferencing, and online communication, navigators or consultants provide patients with individualized assessments of financial difficulties, insurance advice, assistance program matching, application assistance, and ongoing follow-up. This is currently the most widely used intervention in digital health management of the economic toxicity of cancer patients.
(2) Digital screening and automatic referral: Three studies (27, 28, 37) used electronic questionnaires, patient portals, PRO systems or EHR linkage platforms to identify patients’ economic toxicity risks in the early stage, and automatically triggered nurse reminders, navigator intervention or financial service referrals based on preset thresholds, demonstrating the advantages of digital tools in pre-screening and process management of economic toxicity risks.
(3) Insurance navigation and health insurance literacy improvement: three studies (36, 38, 39) helped patients understand insurance terminology, insurance plan structure, medical security policies and related protective regulations through online courses, remote guidance or virtual navigation services, thereby improving patients’ ability to handle medical bills and cope with treatment costs.
(4) Facilitating cost communication and self-management support: one study (32) using the DISCO App to create an individualized question list helps patients prepare for cost-related questions before their visit or provides them with individualized out-of-pocket cost communication before treatment begins, thereby promoting effective communication between patients and clinicians on cost issues, which is an important step in reducing economic toxicity.
(5) Mobile applications or digital platforms: one study (43) use mobile applications or digital platforms for resource matching and assistance access, mainly based on the patient’s clinical information, insurance information or self-reported economic status, to automatically identify available assistance resources and assist in the application.
(6) Telehealth support: two studies (31, 34) adopted a broader approach to telehealth support, reducing healthcare use and indirect economic burden through telehealth or remote care. In addition, Wheeler et al. (26) the core functions and implementation functions of tumor financial navigation intervention were summarized from the perspective of case analysis, providing a basis for the standardization and promotion of digital health financial navigation model.

#### 3.4.2 Intervention implementers

The digital health interventions included in the study exhibited multidisciplinary participation in their implementation, primarily involving financial navigators, financial advisors, social workers, case managers, nursing staff, and clinical research teams that provide services through digital platforms.

(1) Twelve studies (24, 25, 29, 30, 33, 35–38, 40–42) used financial navigators, financial advisors, or case managers as the primary implementers. These studies typically involved specially trained financial navigators, financial advisors, or case managers providing one-on-one financial risk assessments, explaining insurance issues, matching assistance resources, assisting with application processes, and conducting ongoing follow-ups. Overall, the model centered on financial navigators or related professionals is currently the main form of digital health intervention in managing the economic toxicity of cancer patients.
(2) Four studies (23, 27, 34, 44) used nursing staff as the main implementers, suggesting that nursing staff play an important role in digital health intervention and clinical care.
(3) Two studies (32, 43) used digital platforms, mobile applications, or online courses as the main means of intervention, supplemented by professional support. Patients usually receive intervention first through apps, web platforms, online courses, or personalized electronic materials, and then doctors, navigators, or researchers provide answers, communication assistance, or subsequent referrals. Studies have shown that digital tools can not only serve as an auxiliary means of financial navigation, but also become an important entry point for encouraging patients to proactively identify problems, obtain information, and connect with resources.
(4) Four studies (26, 28, 31, 39) place greater emphasis on institutional processes, project implementation or service usage analysis, supplementing the real-world picture of digital health economic toxicity management from the perspective of implementation and service processes.

#### 3.4.3 Intervention phase

Digital health technologies play multiple roles in the management of economic toxicity in cancer patients.

(1) Early diagnosis and treatment: eight studies (24, 25, 33, 35, 36, 40, 42, 44) indicate that identifying patients’ financial risks and providing timely support as early as possible before the economic burden accumulates further is the most common time window for digital health interventions to manage economic toxicity.
(2) Comprehensive treatment period: eight studies (23, 27, 29–32, 37, 43) incorporated digital health interventions into the treatment process and carried out interventions around the patient’s treatment execution, outpatient visits or treatment decision-making process in order to continuously identify and address economic toxicity issues.
(3) Post-discharge follow-up: two studies (38, 41) extended the intervention to the post-discharge, recovery or survivor stage. Patients may continue to experience financial stress, insurance issues and medical burdens. By continuing to implement navigation and insurance support, the long-term financial stress can be alleviated and the ability to use resources can be improved.
(4) Terminal stage: one study (34) focused on terminal or home palliative care for cancer patients. The study supported terminal cancer patients through remote-assisted home palliative care, and the results showed that it could reduce the use of emergency rooms, hospitalizations and ICUs, and reduce related medical insurance expenditures and transportation costs, suggesting that digital health support can be extended to end-of-life care scenarios.
(5) Three studies (26, 28, 39) emphasized the project implementation phase, screening process, or service usage process. The studies suggest that the “intervention phase” of digital health intervention is reflected not only at different points in the patient’s disease development, but also at different stages of the medical system and service process.

### 3.5 Evaluation of the effects of digital health interventions

#### 3.5.1 Economic toxicity and financial difficulties

Eight studies (23–25, 29, 33, 35, 40, 44) have shown that digital health financial navigation interventions have a positive effect on improving the economic toxicity or financial distress of cancer patients. Alacevich et al. (24) pointed out that after point-to-point screening and connection to remote financial counseling, the COST scores of both the individual and group counseling groups improved from the baseline, but the difference between the groups did not reach statistical significance, suggesting that the intervention has preliminary potential in improving economic toxicity, but the efficacy still needs further verification. Bell-Brown et al. (25) used proactive financial navigation on 10 patients with gastric cancer. The results showed that the measure could reduce the proportion of family financial difficulties in newly diagnosed gastric cancer patients within 6 months and reduce the risk of short-term decline in quality of life, but had no significant effect on improving subjective economic stress. Khurana et al. (35) found that after AYA-NAV intervention, the COST score of patients who completed 6 months of follow-up was significantly increased, and economic toxicity was improved. Sadigh et al. (40) found that after CostCOM intervention, the COST score of patients was significantly improved, indicating that individualized cost communication combined with remote financial navigation can alleviate patients’ economic worries. Edward et al. (29) found that in pediatric, adolescent and young adult cancer patients, the psychological response dimension of economic toxicity was improved after FINassist intervention, and the overall economic toxicity level of caregivers also decreased significantly. Song Feifei et al. (23) pointed out that nurse-led navigation services can improve the economic toxicity level of prostate cancer patients and increase their full compliance rate, indicating that nurse-led continuity support has a certain role in alleviating economic toxicity. Henrikson et al. (33) found no significant difference in financial distress and financial well-being compared with enhanced routine care at 12-month follow-up in the CAFÉ randomized trial. Yuan et al. (44) pointed out that although the financial navigation program for breast cancer patients in China improved cost-related health literacy, there was no significant difference in COST-FACIT scores between groups at 1 month, suggesting that the improvement effect of economic toxicity in the short term was limited.

#### 3.5.2 Insurance literacy, cost knowledge and cost communication

Five studies (32, 36, 38, 40, 44) have shown that digital health interventions can improve patients’ insurance literacy, cost knowledge and cost communication skills. Hamel et al. (32) found that after using the DISCO App intervention, cost discussions occurred in all outpatient visits, and patients’ self-efficacy in managing treatment costs and self-efficacy in interacting with doctors were significantly improved, indicating that digital individualized question lists help promote patients’ active participation in cost communication. A randomized controlled trial for AYA cancer patients (36) showed that the HIAYA CHAT intervention significantly improved patients’ health insurance literacy and ACA knowledge and reduced perceived stress at 5-month follow-up, but there was no significant difference in COST scores between groups. Park et al. (38) pointed out that HINT-C significantly improved health insurance literacy and ACA knowledge levels in colorectal cancer survivors, indicating that insurance navigation courses have application value in the long-term financial management of survivors. Sadigh et al. (40) used CostCOM intervention on 20 cancer patients, and the results showed that patients’ economic worries were reduced, and their financial self-efficacy and some health insurance literacy indicators were improved. Yuan et al. (44) pointed out that the financial navigation program can significantly improve the cost-related health literacy of breast cancer patients, indicating that digital education and follow-up support have advantages in improving patients’ ability to cope with medical expenses.

#### 3.5.3 Access to financial assistance, resource referrals and economic benefits

Nine studies (25, 28–30, 35, 37, 40, 42, 43) have shown that digital health financial navigation interventions help improve access to financial assistance, resource referrals, and economic benefits. Lambert et al. (37) found that the TailorMed platform pilot program identified high-priority patients and helped most patients obtain assistance, with a higher cumulative approved savings. Tarnasky et al. (43) found in a time-controlled trial that although the lack of Bridge App follow-up affected the assessment of the primary outcome, an exploratory analysis showed that the intervention group had a higher rate of applying for and receiving financial assistance. Sadigh et al. (40) used the CostCOM intervention, in which a significant proportion of patients completed assistance applications and partially joined the co-payment assistance program. Edward et al. (29, 30) showed that FINassist and CC Links can secure real economic benefits for patients or caregivers. Sadigh et al. (42) reported in a study of brain tumor patients that most financial problems could be resolved through navigation and that some debt relief could be obtained. In addition, Budhu et al. (28) pointed out that there was a significant drop-off from positive screening/request for help to successful contact and record intervention after large-scale screening, with the main obstacle being the inability to contact the patient, suggesting a significant funnel in the referral chain.

#### 3.5.4 Medical use, treatment adherence and cost

Seven studies (27, 31, 34, 40–42, 44) have shown that digital health interventions may indirectly improve economic toxicity-related outcomes by influencing treatment adherence, healthcare utilization, and cost expenditures. Blinder et al. (27) embedded economic toxicity screening into digital PRO monitoring and triggered nurse reminders. The study further suggests that incorporating economic toxicity screening into the digital patient-reported outcome monitoring process can help identify and intervene in financial risks early in clinical care. Jang et al. (34) implemented an intervention in 293 patients with end-stage cancer. The results showed that remote assisted home palliative care can significantly reduce the use of emergency rooms, hospitalizations, and ICUs for end-stage patients, and reduce health insurance expenditures and transportation-related costs. Elshafie et al. (31) found that Telehealth use was associated with better endocrine therapy adherence, but patients had higher out-of-pocket medical costs. In addition, studies (40–42, 44) assessed indicators such as cost-related non-adherence and treatment delays, but the improvement was limited and the conclusions were limited by sample size and follow-up.

#### 3.5.5 Feasibility, acceptability and implementability results

Sixteen studies (24–26, 29, 30, 32, 33, 35–38, 40–44) have shown that digital health financial navigation interventions are generally feasible and well-accepted. Hamel et al. (32) found that implementing it in the outpatient process through the DISCO App was feasible, and patients found it helpful in raising cost-related questions. Khurana et al. (35) used the AYA-NAV intervention method, which had a high acceptance rate and good suitability evaluation among those who tested positive. Park et al. (38) pointed out that the completion rate and satisfaction rate of the HINT-C online insurance navigation course were high, which is consistent with the conclusion of another study (44). Wheeler et al. (26) conducted an in-depth case study on the LIFT financial navigation intervention, which showed that the project has formed relatively clear core intervention and implementation functions, providing a basis for its adaptation and promotion in different cancer care scenarios. At the same time, several studies reported implementation challenges. Alacevich et al. (24) pointed out that the lack of follow-up in trials affected the assessment of the primary outcome, and Tarnasky et al. (43) also reported that the lack of follow-up made it difficult to evaluate the primary economic outcome. Budhu et al. (28) pointed out that “inability to contact patients” is the main obstacle to the implementation of intervention after screening. Some pilot studies showed (41, 42) that the proportion of long-term follow-up or completion of all interventions was limited, suggesting that further optimization of reach and closed-loop management is needed.

#### 3.5.6 Financial problem identification and navigation service utilizes features

One cross-sectional study describes the financial problems exposed and service utilization characteristics of cancer patients using digital navigation services. Patel et al. (39) pointed out that among cancer patients using employer-supported virtual navigation services, most chat logs involved financial issues, including inquiries about expenses, financial concerns, and financial stress affecting physical and mental health. At the same time, patients who reported financial stress were more likely to use services such as concierge referrals, expert opinions, and case management, suggesting that digital navigation platforms have certain value in identifying financial problems and guiding patients to follow-up support services.

## 4 Discussion

Digital health technologies have been extensively studied in the management of economic toxicity in cancer patients. Digital health interventions can be applied to multiple stages of cancer patient diagnosis, treatment, and rehabilitation, especially in the early stages of diagnosis and treatment, and can also extend to the treatment process, survivor follow-up, and end-of-life care. Among these, remote financial navigation and counseling are the most commonly used intervention methods, often implemented in conjunction with components such as digital screening, insurance navigation, and mobile applications. Overall, economic toxicity is associated with outcomes such as financial distress, cost-related non-adherence, and treatment interruption. Future intervention designs need to place greater emphasis on continuous management pathways to achieve more stable and sustainable improvement in the economic toxicity of cancer patients.

### 4.1 The digital health economic toxicity management model is gradually shifting from single intervention to a comprehensive management approach of “screening-navigation-follow-up”

Cancer patients often experience both economic and psychosocial burdens during treatment, and these problems can overlap and influence each other. Economic toxicity is associated with anxiety, depression, and other psychological distress, and may further impact patients’ quality of life and treatment adherence. Digital health technologies offer advantages such as low risk, scalability, and ease of follow-up, and can improve the early identification and accessibility of support services for high-risk patients without significantly increasing their medical burden (45, 46). In recent years, digital health practices in the field of tumor economic toxicity management have gradually shifted from single management to a comprehensive management path of “systematic screening-navigation intervention-continuous follow-up”. Wheeler et al. (47) constructed and tested a multi-stage intervention of standardized economic toxicity screening and one-on-one financial navigation in an academic medical center. After systematic screening, positive patients were included in financial navigation. Through comprehensive assessment and multiple follow-ups, they assisted in completing the application for assistance and progress tracking. The results showed that the COST score was significantly improved after the intervention, and most participants received financial assistance, indicating that applying financial navigation to the care coordination system is expected to reduce the economic toxicity of patients. At the same time, researchers have also begun to shift their focus to the clinical process entry point. Beauchemin et al. (48) evaluated the implementation of the economic distress screening process in outpatient oncology settings, emphasizing the improvement of accessibility and fairness of early identification and early intervention through standardized screening and workflow integration. In terms of intervention models, Mudaranthakam et al. (17) proposed a hybrid financial navigation framework of “technology empowerment + professional human support”, providing an operable design idea for subsequent promotion and scaling in different institutions. In addition, some telemedicine service practices have also begun to incorporate economic toxicity into comprehensive evaluation, suggesting that digital health can not only be used for financial navigation, but also indirectly affect economic stress-related outcomes by reducing travel and medical burdens (49). However, existing evidence is still mainly based on single-center pilot and short-term follow-up studies, focusing on single aspects such as screening, navigation, or access to assistance, and lacking dynamic assessments covering the entire treatment process. Comprehensive management programs for the economic toxicity of cancer patients need to be improved.

### 4.2 Digital health interventions are used in various forms in the management of economic toxicity in cancer patients, but there is significant heterogeneity in the content and implementation of these interventions

(1) The qualifications and role boundaries of the implementers are not clearly defined: Financial navigation intervention involves multiple aspects such as insurance interpretation, cost communication, matching of assistance programs and application assistance. It requires not only a professional understanding of medical insurance policies and billing processes, but also communication, assessment and resource integration capabilities. Existing studies have included financial navigators, social workers, nurses, financial advisors and platform service teams, but different institutions have different definitions of training content, job competency requirements and responsibilities. This may result in significant differences in the actual service depth, types of resources available and follow-up intensity, affecting the replicability and promotion of the intervention (50).
(2) Lack of standardized processes and uniform intervention: Most studies include components such as screening and identification, resource linking and follow-up support, but there are significant differences in key aspects such as screening tools and threshold settings, referral triggering rules, follow-up frequency and duration, degree of digital platform intervention and definition of “closed loop” (51). In addition, the selection of outcome indicators and measurement time points are different, such as COST, financial hardship items, cost-related non-compliance and implementation indicators coexisting, making it difficult to directly compare the effects between different studies (52). In the future, it is urgent to further clarify the core processes, key nodes and evaluation criteria based on existing evidence, and to form a standardized operating procedure and quality control indicator system in order to improve the repeatability and large-scale application of digital health interventions.
(3) Evidence on the long-term effects of digital health interventions remains limited. Cost communication improvement tools, such as the DISCO App, currently mainly show positive short-term results, such as improved cost communication and related self-efficacy. However, whether these benefits can be sustained and further translated into stable improvements in long-term outcomes such as economic toxicity, treatment adherence, and follow-up monitoring adherence still needs to be verified by longer follow-up periods and higher-quality studies.

### 4.3 Personalized digital health interventions offer a new direction for managing economic toxicity

The economic toxicity risk of cancer patients exhibits significant individual differences and stage-specific characteristics. Different patients show marked variations in insurance type, income and family burden, treatment plans, and cost structures, and their economic pressure dynamically changes throughout the treatment process. Therefore, the focus of digital health intervention should shift to personalized digital intervention, namely, achieving precise matching of resources and services based on risk stratification, and continuously adjusting the intensity and content of interventions during follow-up. Current research has attempted to simplify economic toxicity screening tools for application in outpatient settings and to support stratified management (52). Regarding the cost communication process, digital communication facilitation tools can improve communication-related outcomes to some extent, providing an applicable intervention path for on-demand delivery and precise communication (53). At the same time, different patients have different needs for the functionality, usability and delivery methods of digital health tools. Related studies show that intervention design needs to take into account patient preferences, digital literacy and accessibility in order to reduce the digital divide and increase real-world usage (54). The implementation of individualized intervention depends not only on providing services, but also on whether a traceable closed-loop management can be established. That is, by identifying the specific obstacles of patients and improving the efficiency of problem-solving, the process and efficiency can be continuously optimized in a data-driven manner, thereby promoting the stable transformation of “screening-referral-reach-problem-solving” (55). Overall, individualized digital intervention is expected to achieve “serving those who need it most” under limited resource conditions, while improving the efficiency of process improvement to core outcome improvement, and providing a more operable path for the large-scale promotion of digital health economic toxicity management.

## 5 Conclusion

Digital health technologies, with their high accessibility, broad coverage, and lack of time and space constraints, have demonstrated significant value in managing the economic toxicity of cancer patients. This review shows that existing intervention models have gradually shifted from simple education or passive referrals to a comprehensive management path of “systematic screening—financial guidance—continuous follow-up,” showing consistent positive signals in improving cost communication, enhancing insurance literacy, and facilitating access to assistance and resource linkages.

Currently, research in this field is primarily concentrated in the United States, with limited evidence in Asia, indicating an imbalance in digital health research and practice across different healthcare systems. Furthermore, significant differences in intervention content, implementation procedures, and outcome indicators lead to insufficient comparability between studies. Additionally, some studies are limited by sample size, follow-up duration, and reach rates, requiring careful interpretation of conclusions regarding long-term improvements in economic toxicity and systemic cost-effectiveness. Future research should include multi-center, large-sample randomized controlled trials to improve the reliability and generalizability of evidence. Moreover, researchers need to gain a deeper understanding of the dynamic evolution of the economic burden on cancer patients and stratified intervention strategies. Based on screening data, patient-reported outcomes, and healthcare cost information, core risk characteristics and key intervention points at different treatment stages are identified to construct personalized digital intervention plans, thereby improving intervention effectiveness.

In conclusion, digital health interventions are still in a rapid evolutionary stage in the field of cancer economic toxicity management. With the continuous improvement of standardized pathways and the implementation of scientific and health economic evaluations, they are expected to be sustainably promoted in a wider range of clinical scenarios, providing stronger support for reducing the economic burden on cancer patients and their families.

## Data Availability

The original contributions presented in the study are included in the article/supplementary material, further inquiries can be directed to the corresponding authors.

## Glossary

ACA: Affordable Care Act
AYA: Adolescents and Young Adults
COST: Comprehensive Score for Financial Toxicity
COST-FACIT: Functional Assessment of Chronic Illness Therapy-Comprehensive Score for Financial Toxicity
DHI: Digital Health Intervention
DISCO: Discussing Cost
ED: Emergency Department
eFROMs: Electronic Finance-Related Outcome Measures
EHR: Electronic Health Record
FACT-G: Functional Assessment of Cancer Therapy-General
FACIT: Functional Assessment of Chronic Illness Therapy
FN: Financial Navigation
HILM: Health Insurance Literacy Measure
HIAYA CHAT: Health Insurance Education Intervention for Adolescents and Young Adults with Cancer
HINT-C: Health Insurance Navigation Tools for Colorectal Cancer Survivors
HRSN: Health-Related Social Needs
HRSR: Health-Related Social Risk
IARC: International Agency for Research on Cancer
ICU: Intensive Care Unit
JBI: Joanna Briggs Institute
LIFT: Lessening the Impact of Financial Toxicity
NCCN: National Comprehensive Cancer Network
NHI: National Health Insurance
OOP: Out-of-Pocket
PAF: Patient Advocate Foundation
PCC: Population, Concept, and Context
PRO: Patient-Reported Outcome
PROMIS: Patient-Reported Outcomes Measurement Information System
QOL: Quality of Life
QPL: Question Prompt List
RCT: Randomized Controlled Trial
THPC: Tele-Assisted Home-Based Palliative Care
TUQ: Telehealth Usability Questionnaire
